# Salient Points in the Treatment of Catarrhal Deafness1Read at the 35th annual meeting of the Medical Association of Central New York, held at Syracuse, N. Y., October 21, 1902.

**Published:** 1903-01

**Authors:** Sargent F. Snow

**Affiliations:** Syracuse, N. Y., 204 E. Jefferson Street.


### BUFFALO MEDICAL JOURNAL.

VOL. XLII.—LVIII. JANUARY, 1903. No. 6.

### ORIGINAL COMMUNICATIONS.

### Salient Points in the Treatment of Catarrhal Deafness.[1]

BY SARGENT F. SNOW, M. D., Syracuse, N. Y.

IN ADDRESSING this body on chronic deafness, I need offer no apology. Articles on this subject are notable for their absence. We all know this is not because there is lack of material, for there is an abundance of it, and made up of people quite willing and able to have treatment if physicians unite on its advisability.

There is no question but that the successful handling of a case of chronic catarrhal deafness means more careful consideration and more broad painstaking work than any other condition aurists are called upon to treat; obstacles must be recognised and each one overcome before good results are accomplished. What is most needed is that a greater number of the aggressive workers on otology should become more actively interested in this branch of the specialty.

It seems to be generally understood that chronic ear cases are unsatisfactory and beyond improvement, but medical and surgical progress has decreed that an invariably poor prognosis shall not remain the rule. In fact, I believe that a good prognosis should much more frequently be given.

Local intranasal factors must be taken care of before embarking upon a treatment of the deafness itself, but the mistake is often made that they are the whole cause, and too much benefit is expected from their removal. It is many times thought that the hearing will improve or at least by treatment be capable

1. Read at the 35th annual meeting of the Medical Association of Central New York, held at Syracuse, N. Y., October 21, 1902.

of improvement as soon as the morbid nasal states are out of the way, but in 80 per cent. of the cases, this improvement does not come, and why? Simply because there is some unrecognised constitutional factor keeping the eustachian tube occluded, and thereby preventing good returns from the best and most thorough operative work within the nose. A course of treatment attempted before the constitutional factor is removed will prove both unwise and unprofitable.

At present our most effective way of handling chronic cases of catarrhal deafness is by jets of stimulative vapor, made by passing air under pressure over a supersaturated solution of gum camphor in tincture of iodine, through the eustachian tube to the middle-ear, but no good can come from such treatment if the tube is stopped up by congested membranes. No treatment for the relief of chronic catarrhal conditions will be effective if given while the body is covered by an inactive, sensitive skin, causing these congestions.

The common habit of warm bathing, coddling, and living in superheated rooms is most pernicious and sure to be followed by membranes that are thick and loaded; with our modern environment it seems absolutely necessary to do something to counteract these tendencies, hence the observance of personal hygiene becomes one of paramount importance. As a rule cold baths should be taken, but when impracticable, which is seldom, a brisk dry rubbing with a harsh towel not only starts a good reaction, but exercises the skin, an important step in promoting elimination, tone, and vigor.

Either or both of these measures can be depended upon to so toughen the surface that it ceases to act as a menace to the deeper membranes and organs. Head colds are not as common and nature has a chance to produce absorption of inflammatory products.

Protection of the body should become less and less a problem of importance as the patient acquires a prompt skin reaction, but protection for a time has to be considered, and at all times so far as relates to footwear, the feet always needing to be kept warm and dry. Wool or linen mesh are the best materials for undergarments, but they should be of light weight and porous, while the outer clothing should be selected to insure comfort and changed according to the weather conditions.

That pathological states of the liver produce an increase in the thickness of membrane lining the eustachian tube is too often noticed to be gainsayed. In fact, with some a torpid liver is the most prominent constitutional factor, and may block all our efforts to get local treatments through to the middle-ear. A large percentage of these bad chronic cases of deafness can be traced to an over indulgence in the good things of life; the liver becomes unable to do extra work required and curbing the appetite is absolutely necessary.

The stomach and bowels also come in for their share of attention. Dyspeptic or intestinal disturbances, as well as uric acid and uterine troubles produce an irritability of the mucous lining of the head, quite sufficient to necessitate their correction before the tubes remain normally patent. These phenomena of congestion and recongestion of weakened membranes from functional disturbances account in a large measure for the intractibility of chronic catarrhal states.

Deafness in the subacute form, or in recent cases may respond to rather crude method of treatment, but when we meet the more aggravated forms, some of which have been slowly progressive for ten, or twenty years, we have a different proposition before us, and but few specialists without an experience in general practice are equipped for the work, and not even then unless they be painstaking and persistent. Every little side issue must be studied to see what influence it may have on the ear condition.

Habits and environment of the chronically deaf need most careful scrutiny, for they must be corrected or it is better to advise the patient against further treatment. Professional reputation is too precious to be jeopardised by an attempt to handle such cases without the intelligent and willing coöperation of all intimately concerned.

Vigorous exercise can be made a great aid in relieving distended blood-vessels of their load. It has been the author's frequent experience to find it impossible to keep the eustachian tubes clear until the patients were persuaded to do vigorous active arm exercise every morning as a routine. Local treatments do little more and are not always within reach.

Fleshy people are prone to avoid exertion, but they are not the only ones who need urging to physical exercise. We find them in all lines of clerical or professional work. These people practically use nothing but their brains; all their head membranes are in a state of passive congestion with dilated blood-vessels, which condition may be effectively relieved by vigorous arm movements. Surgical and therapeutic problems are small compared with, or difficulty in, regulating the diet.

Cold bathing as a requirement is not such a hardship after a little practice, neither must one have modern bath conveniences; a wash basin will contain enough water to start a good reaction, and if used immediately after the exercises above mentioned will materially assist in starting the mucus and contracting the arterioles. The dry rub down at night acts in a similar manner and prevents taking cold after perspiring.

It goes without saying that necessary operative work within the nose must be done and done thoroughly; it is one of the "points of necessary prominence." No one with a projecting nasal spur, pressing middle or prolapsed lower turbinate can avoid frequent congestions; but experience in otological work has proved to me that the reason why many attempts to relieve chronic catarrhal deafness result in failure is principally because the constitutional factors have not been appreciated. Treatments have been forced through an eustachian tube still too sensitive, only to be followed by an unpleasant reaction, and an increase in the bad symptoms.

A manifest error is that argument that because the patient has increased deafness after a treatment he cannot be helped. Experience shows that either this increased deafness will clear up with a few daily trials or that it is simply an evidence that our attempt at improving the function is too previous. All causative factors have not been gotten out of the way or enough time has not elapsed since our operative work for nature to readjust herself to the new conditions, the eustachian membranes are still so sensitive that even the injection of a mild vapor causes them to become more swollen, which effect later on, under a better local and constitutional state, will not be produced.

To sum it all up it is virtually impossible to successfully handle a case of chronic catarrhal deafness where there is low vitality, a sensitive skin, or frequent functional derangements; whereas, without these obstacles, a correction of morbid nasal conditions makes the disease non-aggressive and capable of much improvement by the persistent camphor and iodine vapor treatment.

204 E. Jefferson Street.

